# Supplementary material for: Stability Study of Parenteral N-Acetylcysteine, and Chemical Inhibition of Its Dimerization
Source: Pharmaceuticals (Basel). 2023 Jan 3;16(1):72. doi: 10.3390/ph16010072 (PMC9860916; doi:10.3390/ph16010072)

Supplement Material

I. Chromatograms of N-Acetylcysteine forced degradation

Figure S1. Heat (80 ^{o}C, 3 h)

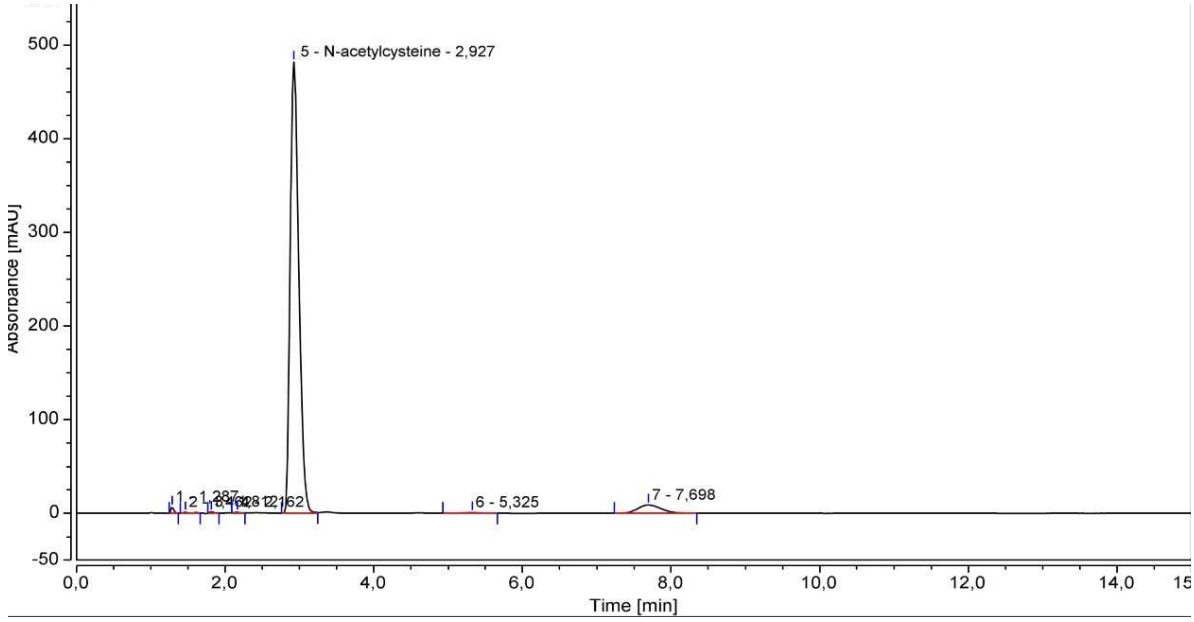

Figure S2. Light (sunlamp, 28 days)

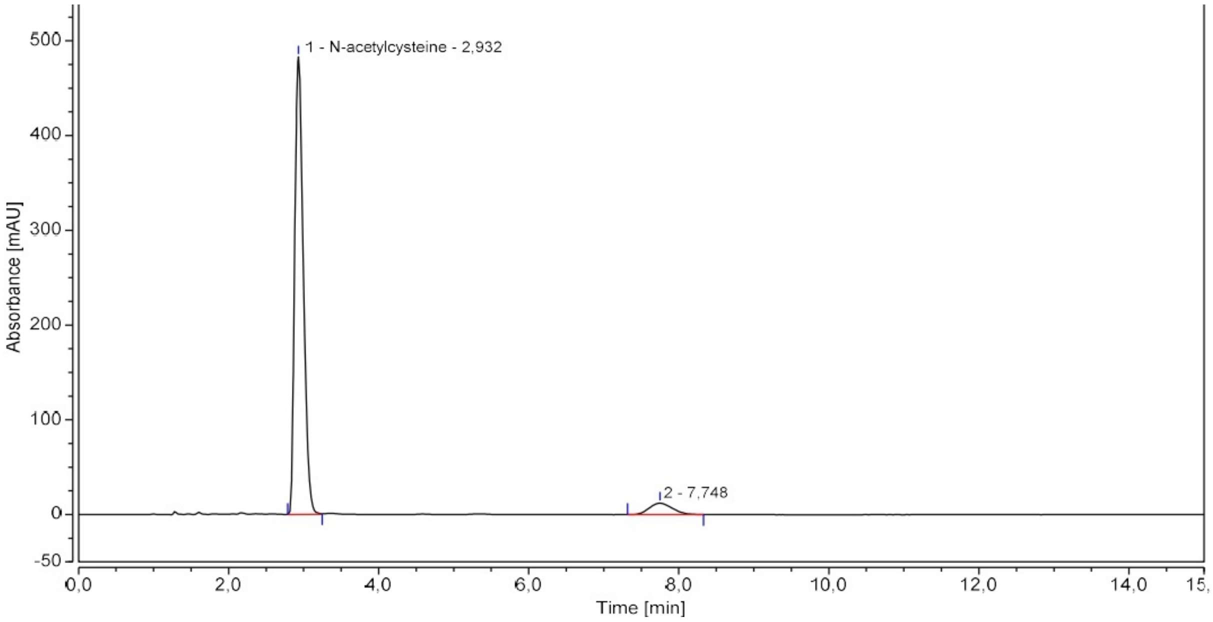

Figure S3. Oxidation (H_2O_2 0.3 %, 3 h)

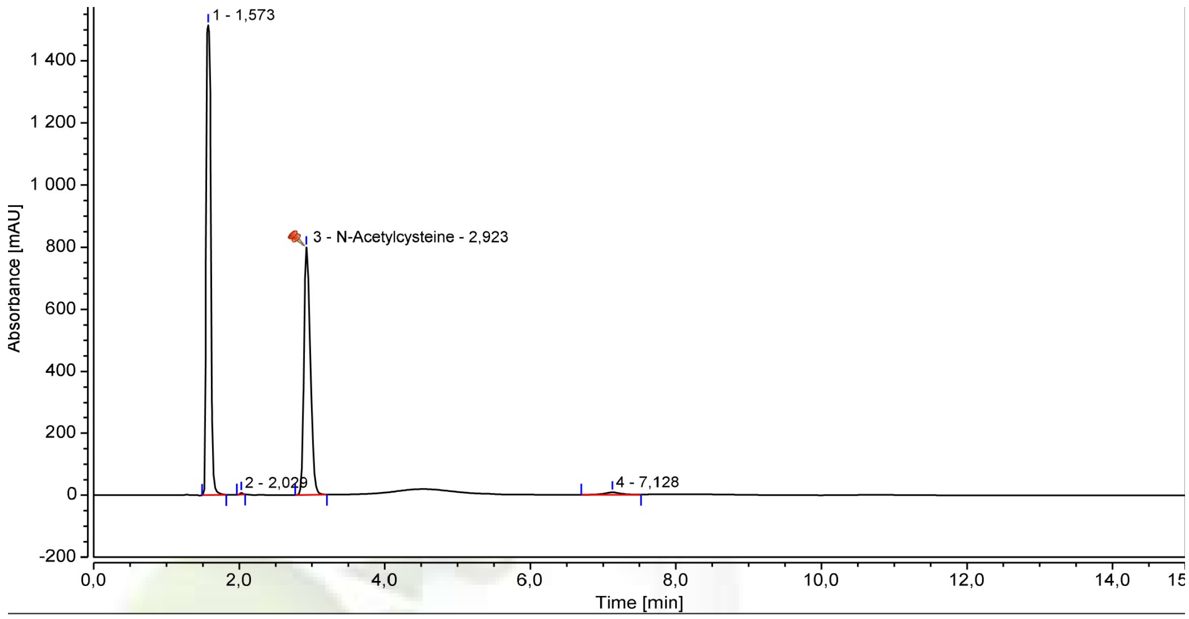

Figure S4. Acid (HCl 0.5 M, 1 min)

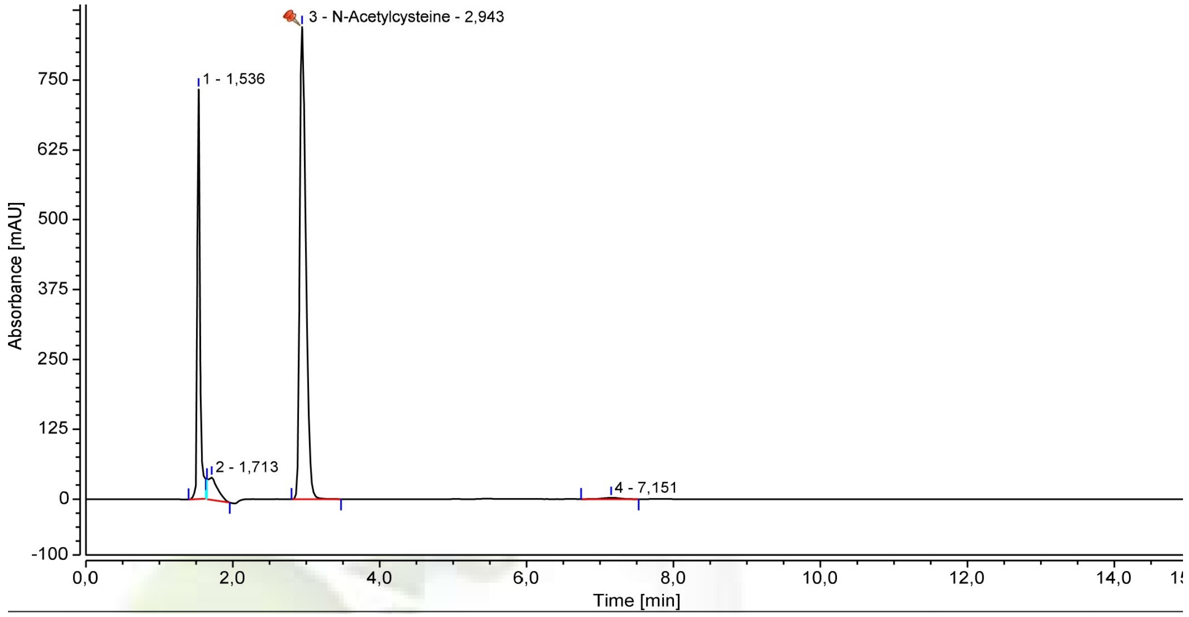

Figure S5. Alkaline (NaOH 0.1 M, 10 min)

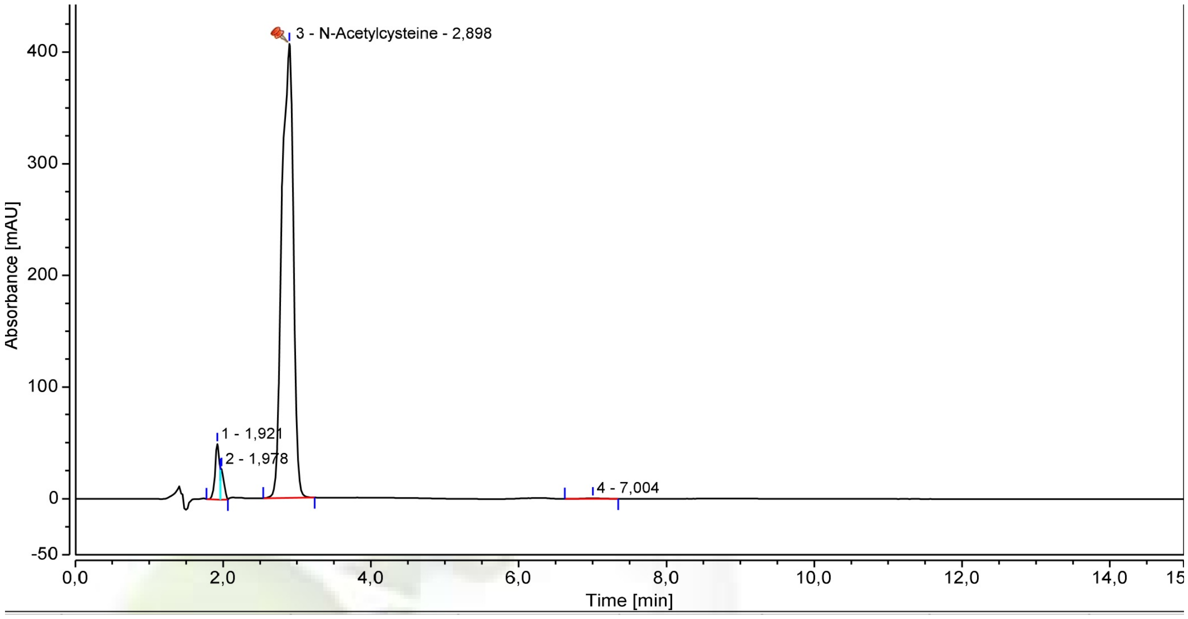

II. Chromatogram of N-Acetylcysteine dimer

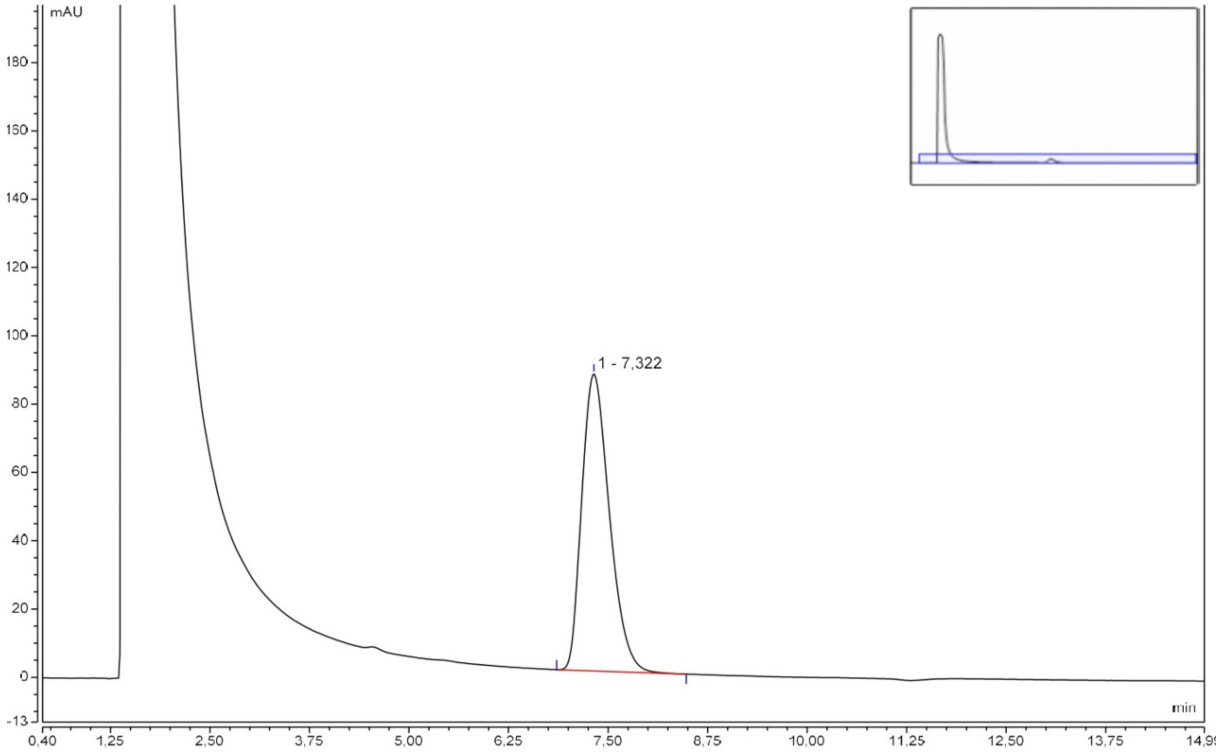

Supplement: Supplementary file 1 [file pharmaceuticals-16-00072-s001.zip › pharmaceuticals-2080222-supplementary.pdf]
